# Extraction method shapes soil water-soluble organic matter composition as revealed by absorbance, fluorescence, and parallel factor analysis (PARAFAC)

**DOI:** 10.1038/s41598-026-41455-w

**Published:** 2026-03-07

**Authors:** Christina Fasching, Kyle S. Boodoo, Annika Feld-Golinski, Mansour A. Foroushani, Peter Chifflard

**Affiliations:** 1 Department of Geography, Philipps-University Marburg, Marburg, Germany; 2 Department of Geography and Regional Research, University of Vienna, Vienna, Austria; 3 Kompetenzzentrum Wasser Hessen, Frankfurt, Germany; 4 Environment and Climate Research Hub (ECH), University of Vienna, Vienna, Austria

**Keywords:** Water-soluble organic matter (WSOM) extraction, Dissolved organic matter (DOM) composition, Dissolved organic carbon (DOC) concentration, Potassium sulphate (K_2_SO_4_), Parallel factor analysis (PARAFAC), Excitation-emission spectra (EEM), Biogeochemistry, Ecology, Ecology, Environmental sciences

## Abstract

Organic matter (OM) is central to biogeochemical processes in both soils and aquatic systems. Water-soluble organic matter (WSOM), leached from soil, is widely analyzed as a proxy for the mobile OM fraction, yet the chemical composition of extracts depends strongly on the extraction method used. We compared two WSOM extraction protocols—distilled water and 0.5 M K_2_SO_4_—across 217 soil samples from 83 depth profiles spanning four central European regions. Absorbance and fluorescence spectroscopy with PARAFAC modeling were used to characterize dissolved organic carbon (DOC) concentration and composition—approaches increasingly applied in soil science to trace soil organic matter dynamics. DOC generally declined with profile depth. K_2_SO_4_ extracts consistently yielded higher DOC concentrations, dominated by humic-like fluorescence. Water extracts were more variable, with stronger protein-like signals—showing clearer depth-related trends, with deeper layers enriched in microbially-derived DOM. This higher variability likely reflects the dynamic nature of labile WSOM fractions. We highlight the importance of extraction chemistry: water-based methods capture reactive, microbially-produced WSOM—likely indicators of immediate inputs to aquatic systems, whereas salt-based methods emphasize more stable pools—acting as indicators of less bio-available, long-term terrestrial reservoirs. Extraction methodology selection should consider the study objectives and specific biological and physicochemical processes investigated.

## Introduction

Soil organic matter (SOM) plays a central role in regulating biogeochemical processes in terrestrial and aquatic ecosystems, influencing nutrient cycling, microbial activity, and carbon fluxes^1,2^. It comprises a continuum of compounds of varying size and degrees of decomposition and humification, originating from plant and animal residues, as well as microbial products^3^. SOM can be found in particulate form, bound to mineral surfaces, or as dissolved organic matter (DOM) in soil pore water. The soil DOM pool receives continuous inputs from fresh organic matter such as plant litter, root exudates, and microbial products, while simultaneously undergoing chemical and microbial transformations that alter its composition along soil horizons^4,5^. The extractable fraction of SOM (extracted using water or dilute salt solutions) is typically referred to as water-soluble organic matter (WSOM)^6^, and represents a small but functionally important component of SOM that is frequently used to trace soil DOM. WSOM chemical characteristics influence processes such as metal transport and bioavailability^7^. Mobilized by infiltrating water, WSOM can be temporarily stored, transported through soil profiles, and eventually exported to aquatic systems^4,5,8^, linking terrestrial and aquatic carbon cycling. However, tracking DOM dynamics in soils remains challenging due to methodological constraints such as cost and time requirements^4^. In this context, absorbance and fluorescence spectroscopy are increasingly employed as valuable tools to characterize WSOM composition and help trace its transformations and transport within the soil–water continuum.

Absorbance and fluorescence spectroscopy are powerful, low-cost tools for characterizing organic matter, due to their sensitivity and speed. They are widely applied to trace DOM in aquatic systems^9–11^, and their potential for soil DOM is being increasingly recognized, with several studies demonstrating their utility^12–16^. Given these advantages, such techniques hold promise as tracers of DOM dynamics in soils, where they can help to uncover key processes that still remain poorly understood.

A key challenge for the application of absorbance and fluorescence spectroscopy in tracing DOM in soils, however, lies in the lack of standardized protocols for extracting water-soluble organic matter (WSOM). Extraction chemistry strongly shapes the quantity and composition of dissolved organic carbon (DOC) and nutrients, making cross-study comparisons using different extractants and methodologies difficult^12,17^. For instance, hot-water extraction is often used to maximize the recovery of labile organic matter for detailed chemical characterization^18,19^, while cold-water or ultrapure water extractions (e.g. Milli-Q) tend to isolate more hydrophilic and protein-like compounds^20^. Other approaches include the use of CaCl_2_ solutions to extract DOC and dissolved nitrogen from soil profiles^21^ or use of potassium sulfate (K_2_SO_4_), which has been preferred to reduce metal interactions, and yield more realistic SUVA_254_ values^12^. Recent studies using ultrapure water extractions and a short shaking period at room temperature revealed distinct WSOM patterns across diverse agricultural regions^13,22^ and soils in China^23^. Another study^12^ showed that WSOM extraction using salt solutions resulted in higher fluorescence index and humification index values, compared to when extraction was done with water only, and that there were distinguishable differences between the fluorescence signal of different salts. Together, these examples illustrate that WSOM and dissolved nutrient concentrations vary substantially with extraction method, underscoring the need for careful protocol selection.

Here, we compare two commonly used water-soluble organic matter (WSOM) extraction methods— the use of (i) distilled water (*Aqua dest.*) and (ii) 0.5 M potassium sulfate (K_2_SO_4_) solution. Samples were obtained from a broad range of Central European landscapes: the Tyrolean Alps (Austria), and the Ore Mountains, Sauerland, and the Black Forest (Germany). Comparison of sample results between sites of differing geology, as well as over individual depth profiles, allows for the determination of the validity and transferability of the findings. Given the potential of spectroscopic techniques such as absorbance and fluorescence to provide rapid and cost-effective insights into WSOM dynamics, we evaluate both extraction methods based on established optical indices, including: SUVA_254_, the humification index (HIX), and the biological index (BIX). In addition, we applied parallel factor analysis (PARAFAC) to model fluorescent components and assess WSOM compositional differences. This comparison aims to inform best practice for characterizing WSOM concentration and composition (based on absorbance and fluorescence), depending on the application.

## Methods

### Site description and sampling methodology

We sampled several locations in the Tyrolean Alps in Austria (14 sites), as well as the Ore Mountains (12 sites), Black Forest (37 sites) and Sauerland (20 sites) in Germany. In the Sauerland (Germany), sampling focused on the Obere Brachtpe catchment (2.6 km^2^; gauge Husten), a tributary of the larger Hüppcherhammer basin (47.2 km^2^) within the Rhenish Massif. Elevations range from 313 to 514 m a.s.l., and the landscape is characterized by a mix of pasture, spruce plantations, and mixed forest. The climate is temperate, with an average annual temperature of ~ 9.1 °C and precipitation of ~ 1227 mm, of which 15–20% typically occurs as snowfall. Loamy Cambisols derived from periglacial slope deposits complemented by Leptosols and Stagnosols are the most prominent soils in the catchment.

The Padasterbach catchment in the Austrian Tyrolean Alps (11.2 km^2^) drains into the River Sill, within the Wipp Valley. Elevation spans from the valley floor at ~ 1060 m a.s.l., up to the Schafseitenspitze summit at 2602 m a.s.l. Vegetation includes alpine pastures and dwarf shrub communities in the upper reaches, transitioning to montane spruce forests downslope. The catchment lies within the metamorphic zone of the Central Alps, with the dominant soil types being podzolic Cambisols and Podzols, transitioning to Leptosols with shallow A–C profiles due to limited pedogenesis on the steep, rocky terrain of the high alpine environment.

In the Black Forest region of south-western Germany, we studied the nested Rütlibach (0.21 km^2^; 340–585 m a.s.l.; 47.9570° N, 7.8378° E) and Eberbach (1.5 km^2^; 300–640 m a.s.l.) catchments, both located within the Dorfbach basin near the western margin of the forest. Crystalline bedrock, overlain by periglacial drift, dominates the geology. Cambisols have developed in the periglacial drift cover and the soil texture is dominated by sandy loam, with the forested upper slopes giving way to grasslands at lower elevations.

In the Ore Mountains (Germany), we examined the upper Freiberger Mulde catchment (77 km^2^), which ranges in elevation from 446 to 850 m a.s.l. The region experiences an average annual temperature of 6.6 °C and approximately 930 mm of precipitation per annum. The terrain is defined by elevated plateaus and steeply incised valleys. The dominant geology includes gneiss and phyllite beneath periglacial cover layers, with the prevailing soil types being Stagno-Gleyic Cambisols and Dystric Cambisols. Land use is primarily spruce forest (50%), with pasture (25%) and cropland (20%) also present.

**Fig. 1:**
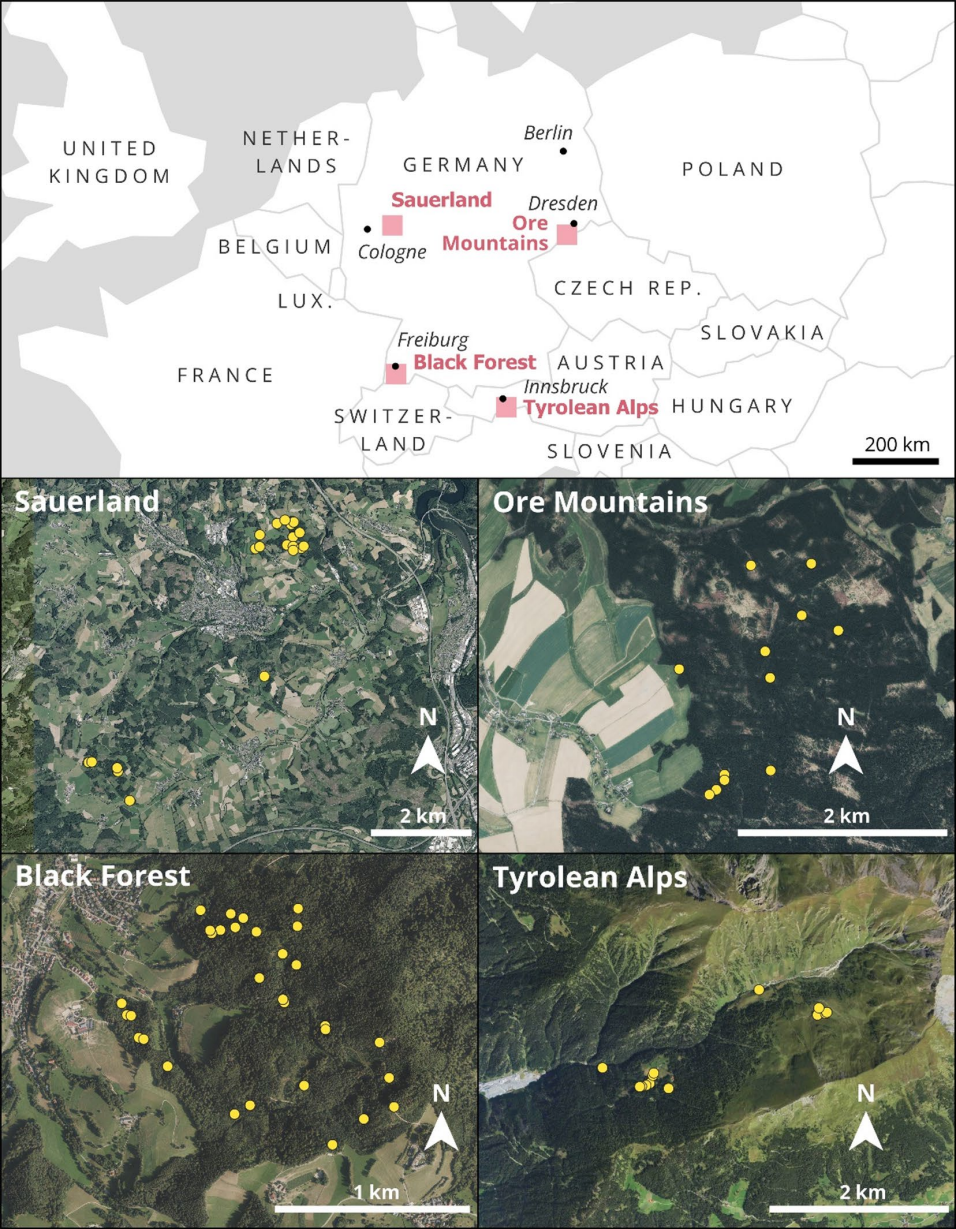
Visualization of the sampling locations in the four study regions where soil cores were extracted: Sauerland, Ore Mountains, Black Forest, and Tyrolean Alps^24^. The visualization was created by Christiane Enderle, Philipps-University Marburg, using QGIS 3.40.8 Bratislava (https://qgis.org/download/, last access 2026-02-18). The overview map is based on Natural Earth data (public domain; https://www.naturalearthdata.com/about/terms-of-use/, last access 2026-02-18; https://www.naturalearthdata.com/downloads/10m-cultural-vectors/ and https://www.naturalearthdata.com/downloads/110m-cultural-vectors/, last access 2026-02-18). Orthophotos: Sauerland: © Nordrhein-Westfalen 2026, dl-zero-de/2.0, Datenlizenz Deutschland—Zero—Version 2.0 (https://www.govdata.de/dl-de/zero-2-0), Digitale Orthophotos (JPEG2000, 10-fache Kompression)—Paketierung: Einzelkacheln (https://www.opengeodata.nrw.de/produkte/geobasis/lusat/akt/dop/dop_jp2_f10/, last access 2026-02-13). Ore Mountains: © Geodaten Sachsen 2026, dl-de/by-2-0, Datenlizenz Deutschland—Namensnennung—Version 2.0 (https://www.govdata.de/dl-de/by-2-0), WMS Digitale Orthophotos (RGB) (https://www.landesvermessung.sachsen.de/luftbild-produkte-4982.html, last access 2026-02-18). Black Forest: © LGL 2026, dl-de/by-2-0, Datenlizenz Deutschland—Namensnennung—Version 2.0 (https://www.govdata.de/dl-de/by-2-0), Digitales Orthophoto DOP 20 (https://opengeodata.lgl-bw.de/#/(sidenav:product/dop20), last access 2026-02-12). Tyrolean Alps: © Land Tirol 2026, CC BY 4.0 (https://creativecommons.org/licenses/by/4.0/ ), Orthofoto Tirol (https://www.data.gv.at/datasets/35691b6c-9ed7-4517-b4b3-688b0569729a?locale=de, last access 2026-02-18).

Sampling was conducted during the period: March - August 2023. Within each catchment, we extracted soil depth cores using an automatic percussion drill (Makita HM1512 AVT). Soil sampling for WSOM analysis was guided by approximate depth intervals (targeting ~ 1–10, 11–50, 51–100, 101–200 cm), but actual sampling depths were adjusted to soil horizon boundaries at each site; samples were subsequently grouped into representative depth classes for analysis (total: 217 soil samples collected from a total of 83 soil cores; Fig. 1). Soil sub-samples (composite horizon specific samples) were placed in labelled plastic bags, excess air removed, the bag sealed and stored in a cooling box at 4 °C in the dark, until transported to the laboratory of the Department of Geography, Philipps-University of Marburg. Soil samples were then kept frozen at − 20 °C until further processing 25.

### Sample preparation and field parameters

We compared two different extraction methods for soil samples, either using distilled water (Aqua dest.) or potassium sulphate, K_2_SO_4_. For both methods, 40 g of unsieved field-moist soil (composite samples), with large sediment and plant fragments ( ~ > 4 mm) removed, was defrosted at 4 °C overnight to minimize microbial degradation. Each soil sample was thoroughly mixed and then split into two identical sub-samples, which were placed in separate 1 L pre-combusted glass bottles, together with either 160 mL Aqua dest. or 0.5 M K_2_SO_4_^17^. Samples were placed on a shaker Table (200 revolutions/minute) for 1 h, under dark conditions, and at 4 °C to limit potential microbial degradation of DOM. The soil leachate was decanted, centrifuged, and filtered through a double layer of pre-combusted (450 °C) glass fiber filters (Whatman GF/F, pore sizes 0.7 μm). This protocol was adapted from a protocol originally done at room temperature^12^; however, we opted for 4 °C to limit microbial degradation. By splitting each composite (mixed soil) sample, we ensured that both extraction methods were applied to identical material, allowing direct comparisons of DOC concentration and composition.

Filtered water samples were stored in 40 mL glass vials (pre-soaked in 0.1 N HCl, rinsed thoroughly with ultrapure water (Milli-Q water) and combusted for 4 h at 450 °C), sealed with Teflon-coated septa (pre-soaked with 0.1 N NaOH and rinsed thoroughly with Milli-Q water) and stored at 4 °C in the dark until measurement (within hours after extraction).

### Laboratory analyses

The DOC concentration of the filtered samples was measured using a TOC analyzer, via high-temperature combustion of organic matter, followed by thermal detection of CO_2_ (TOC-L, Shimadzu, Japan—detection limit, 4 µg L^−1^). Process blanks of the entire workflow using Aqua dest. and K_2_SO_4_ were employed. Fluorescence analyses were performed on a fluorescence spectrometer (RF-6000, Shimadzu). Excitation-emission matrices (EEMs) were generated by measuring fluorescence intensities at excitation wavelengths ranging from 200 to 450 nm (5 nm increments) and emission wavelengths from 250 to 700 nm (2 nm increments) with a scan speed of 12,000 nm min^−1^ and using a 1 cm quartz cuvette, in accordance with the methodology outlined previously^26^. Absorbance was measured with a UV–VIS spectrophotometer (Genesys 10 S, ThermoFisher), using 10 cm quartz cuvettes. Similar to the DOC analysis, process blanks of the entire workflow were determined using Aqua dest. and K_2_SO_4_.

### DOM absorbance, fluorescence, and parallel factor analysis (PARAFAC)

The following indices were calculated based on absorbance measurements: The Specific UV Absorption at 254 nm (SUVA_254_) was calculated as the absorption coefficient at 254 nm (m^−1^) relative to the DOC concentration (mg L^−1^)^27^. This index was reported to correlate positively with increasing DOM aromaticity. The absorption coefficients at 254 nm (m^−1^) (a254) and 300 nm (m^−1^) (a300) can be used as an indicator for chromophoric DOM ^28^. The slope ratio (SR) was computed as the ratio of S275–295 to S350–400; this parameter is reported to correlate inversely with DOM molecular weight^29^.

Excitation–emission matrices (EEMs) were corrected for the inner filter effect using absorbance measurements. Prior to PARAFAC modeling, EEMs were preprocessed following standard procedures implemented in the R package *staRdom*^30^. This included blank subtraction, removal of first- and second-order Rayleigh and Raman scatter (± 20 nm around the scatter bands), and interpolation of the removed regions using a spline interpolation. Instrumental noise at the edges of the excitation and emission ranges was excluded, and EEMs were Raman-normalized using the area under the Raman peak of Milli-Q water. Individual fluorescent components were modeled using parallel factor analysis (PARAFAC). Models with 3–12 components were calculated using non-negativity constraints on excitation, emission, and sample modes, and F_max_ normalization was applied. The global minimum of model error was assessed using 50 random starts for each model. The optimal model was selected based on residual analysis, visual inspection of excitation and emission loadings, and split-half validation. Sample and wavelength leverage diagnostics were used to identify outliers which were excluded from the final model.

**Fig. 2:**
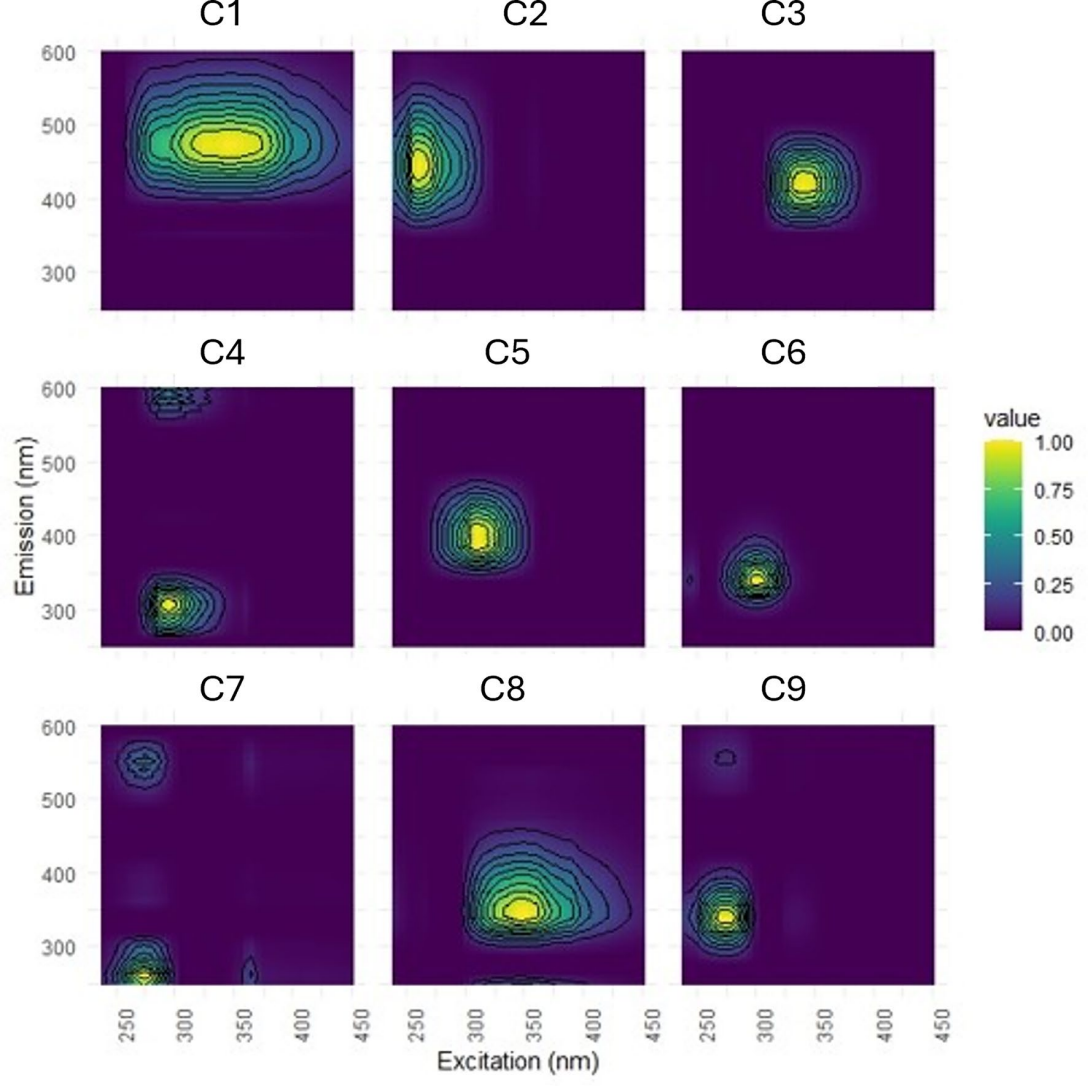
Excitation and Emission maxima of the 9 fluorescent components modeled by parallel factor analysis (PARAFAC). Components C1–C3 and C5 were assigned humic-like, and C4 and C6–C9 were assigned protein-like based on closest matches with the OpenFluor Database^34^ (see Table 1).

The final PARAFAC model resolved nine fluorescent components: C1–C3 and C5 resembling humic-like fluorescence, and C4 and C6–C9 resembling protein-like fluorescence (Fig. 2; Table 1). All components were standardized to total fluorescence prior to further analysis.

Additionally, based on the EEMs, we calculated the humification index (HIX), an indicator of DOM aromaticity, as the fluorescence intensity in the 300–345 nm region divided by the sum of intensity in the 300–345 nm and 435–480 nm regions^31^. Higher values correspond to lower H:C ratios, and indicate a higher extent of humification. The Biological index (BIX), an indicator of autochthonous biological activity, was calculated by dividing the fluorescence intensity emitted at 380 nm, by the fluorescence intensity emitted at 430 nm, both at an excitation of 310 nm^32^. All statistical analyses and visualizations were performed within the R statistical environment^33^.

**Table 1:** Excitation (Ex) and Emission (EM) maxima of the 9 fluorescent components modeled by parallel factor analysis (PARAFAC). Components C1–C3 and C5 were assigned humic-like, and C4 and C6–C9 were assigned protein-like based on closest matches with the OpenFluor Database^34^. While component C4 and C8 could not be matched with the database, they resembled protein-like fluorescence with emission maxima under 400 nm^35^.

Component	EX	EM	Description	Matching component and citation
1	345	474	Terrestrial humic-like	C2^36^
2	260	448	Terrestrial humic-like, aromatic intermediate MW	C4^37^
3	340	416	Humic-like, Low aromaticity, low MW, Fulvic-like	C5^37,38^
4	295	308 (600)	Protein-like	^35^
5	310	406	Marine humic-like associated with biological activity. Also found in catchments under agricultural influence	^34^,C3^37^
6	300	340	Microbial-like, microbial activity in springs after biological input	C3^40^
7	275	262 (540)	Protein-tyrosine-like	^34^,C6^39^
8	350	346	Protein-like	^35^
9	275	340	Protein-like (Tryptophan-like), indicating biological activity	^34^,C1^40^

### Data analyses

We tested the effect of the two extraction methods (Aqua dest. and K_2_SO_4_) on dissolved organic carbon (DOC) concentration and dissolved organic matter (DOM) composition using paired *t*-tests. In addition, a principal component analysis (PCA) was conducted to visualize differences in DOM composition between the two extraction methods. All statistical analyses were performed in the R statistical environment^33^.

## Results

### DOC concentration

**Fig. 3:**
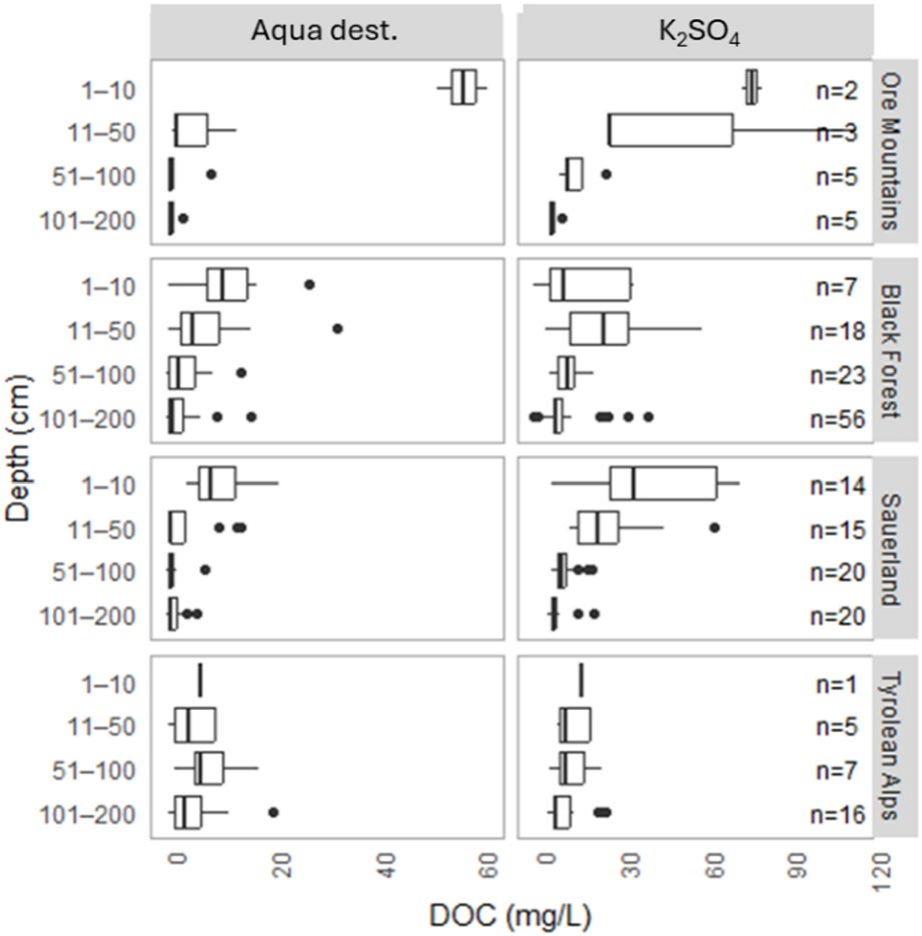
Boxplot of DOC concentration along depth (cm) across all 4 regions based on both extraction methods (distilled water (Aqua dest.) and K_2_SO_4_).

Soil DOC concentration differed significantly based on the extraction method used (paired t-test, *p* < 0.001, *n* = 217). DOC extracted with Aqua dest. averaged 6.82 ± 11.67 mg C L^− 1^ and ranged from 0.01 to 180.1 mg C L^− 1^, while DOC concentrations extracted using K_2_SO_4_ averaged 16.79 ± 16.5 mg C L^− 1^ and ranged from 0.01 to 115.3 mg C L^− 1^. DOC concentrations typically decreased with depth, regardless of the extraction method used, except in the Tyrolean Alps, a site with predominantly Lithic and Rendzic Leptosols, having only a thin sediment layer above the bedrock material, where concentrations remained relatively constant throughout the soil profile (Fig. 3).

### DOM composition

PARAFAC modeling identified a total of 9 distinct fluorescent components (Fig. 2; Table 1), with Components C1-C3 resembling humic-like terrestrial fluorescence often found in rivers^39^, and component C4 and C6–C9 matching widely reported protein-like components^35,40^, C5 has been associated with microbially derived humic-like material found in catchments with agricultural influence^39^. The spectra of components C4 and C8 did not show a strong match with any fluorescence component published in the Open-Fluor database, but their fluorescence was comparable to protein-like fluorescence^35^.

**Fig. 4:**
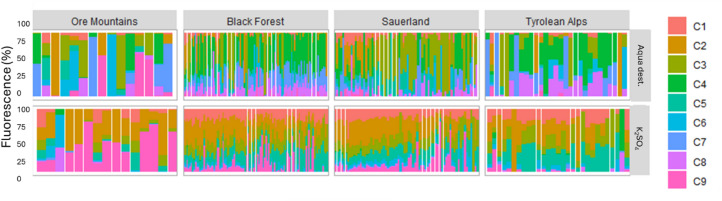
Bar chart of WSOM composition (PARAFAC components in percent of total fluorescence) of all samples for both extraction methods. Samples are ordered according to sample depth in the individual plots. Components C1-C3 and C5 were assigned as humic-like, and C4 and C6-C9 were assigned protein-like based on closest matches with the OpenFluor Database^34^ (see Table 1).

**Fig. 5:**
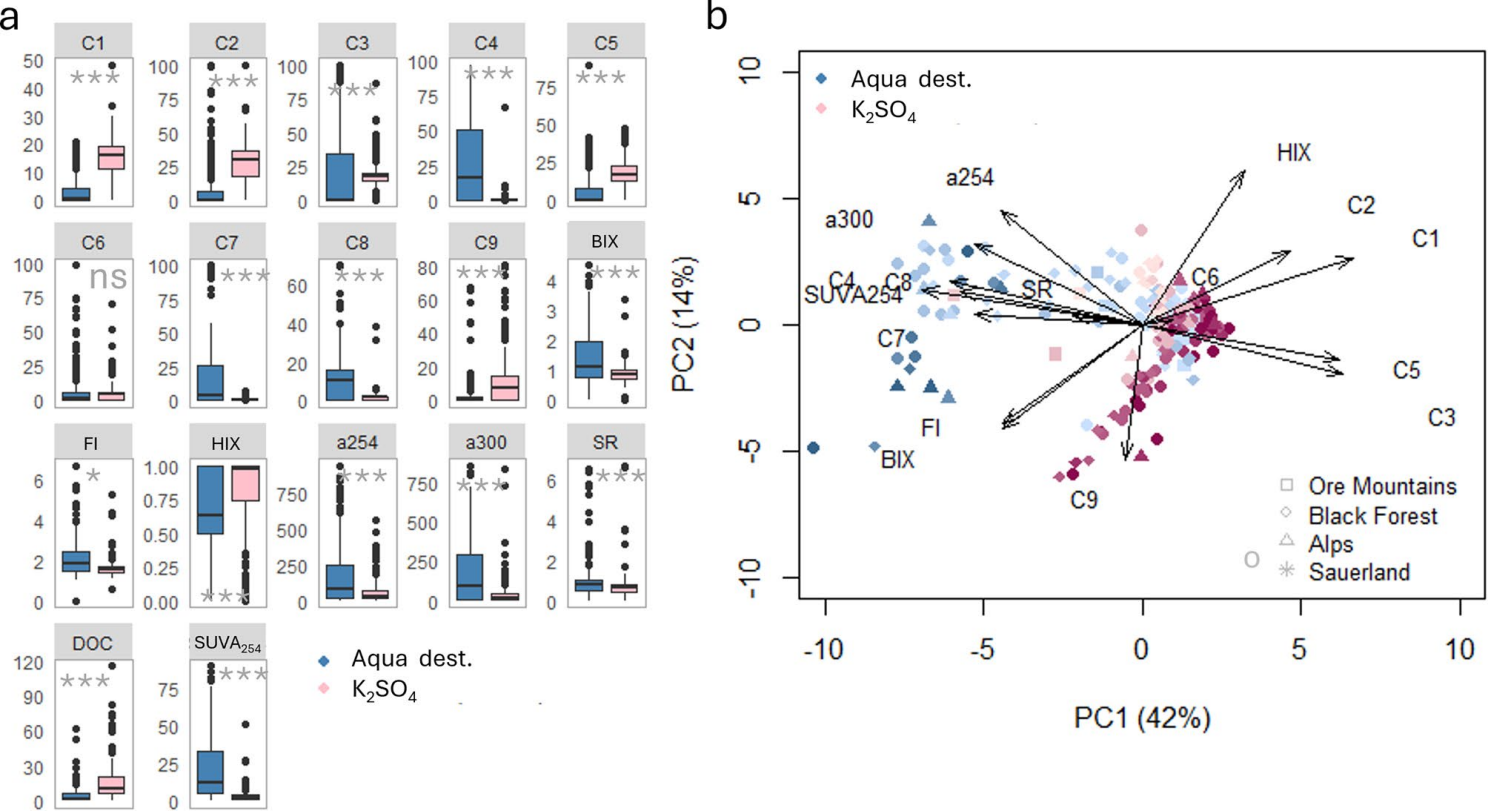
WSOM concentration (DOC; mg l^− 1^) and composition across catchments based on absorbance and fluorescence indices. Shown are the humification index (HIX) and biological index (BIX), as well as humic-like (components C1–C3, C5) and protein-like (components C4, C6–C9, in % of total fluorescence) fluorescent components (see Table 1) for both extraction methods. (**a**) Asterisks in boxplots indicate significance levels based on paired t-tests (ns: not significant, *: <0.05, **: <0.01,***: <0.001). (**b**) Arrows in the PCA represent structure coefficients, with colors indicating extraction methods. Color intensity represents sample depth, with lighter shades indicating surface and near-surface layers, and increasingly darker shades indicating increasing depths.

We found WSOM composition (based on absorbance and fluorescence measurements) to differ significantly between the extraction methods (Figs. 4 and 5a, paired t-test, *p* < 0.001, *n* = 217). Overall, the protein-like contributions (sum of all protein-like components) were markedly higher in the Aqua dest. extracts (61 ± 39%) compared to the K_2_SO_4_ extracts (21 ± 19%) (t-test, *p* < 0.001, *n* = 217). Principal component analysis (PCA) comparing the K_2_SO_4_ and Aqua dest. extracted soil samples revealed a clear separation of the samples analysed with the different methods (Fig. 5b). The K_2_SO_4_ extract samples clustered distinctly, characterized by elevated contributions of humic-like fluorescence C1, C2 and C3, with little contribution of the protein-like fraction (C5). On the other hand, the Aqua dest. extracted WSOM was characterized by higher contributions of protein-like fluorescence (C4, C8) and higher biological activity (as indicated by higher values of BIX), but also more chromophoric and aromatic WSOM (as indicated by higher values of SUVA_254_ and a300).

**Fig. 6:**
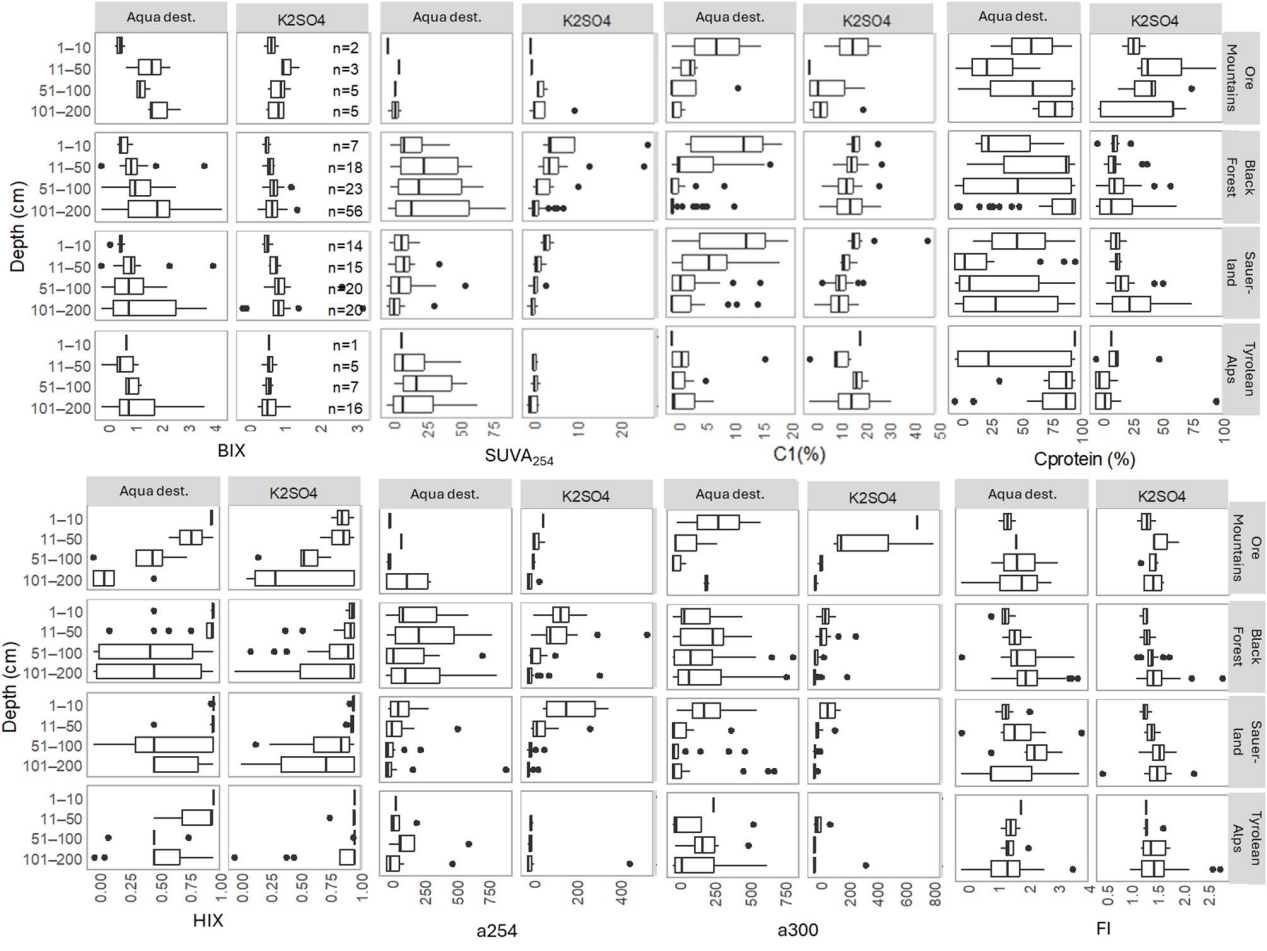
WSOM composition across catchments based on absorbance and fluorescence indices: biological index (BIX), Specific UV Absorbance at 254 nm (SUVA_254_), as well as humic-like (components C1) and protein-like fluorescence (C_protein_) humification index (HIX), absorbance at 254 and 300 nm and the Fluorescence index (FI) for both extraction methods.

**Fig. 7:**
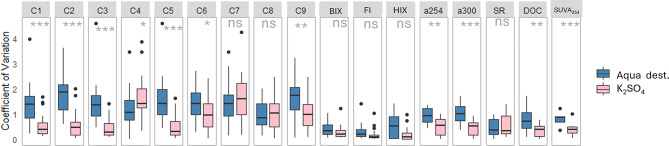
Boxplots of the coefficient of variation of WSOM composition. Shown are PARAFAC components in percent of total fluorescence and indices (HIX, FI, BIX, a254, a300, SUVA_254_) of all samples for both extraction methods along the depth profiles. Asterisks indicate significance levels based on paired Student’s t-tests (ns: not significant, *: <0.05, **: <0.01, ***: <0.001).

Depth gradients were more pronounced when using the Aqua dest. extraction method (Figs. 5b and 6) with a general transition from more humic and terrestrially derived contributions in surface layers to more protein-like contributions in deeper layers (Fig. 6). In the Ore Mountains and Sauerland, there was an initial spike in proteinaceous fluorescence in the surface layer (Fig. 6). Across all regions, the BIX increased with depth. In contrast, the K_2_SO_4_ extracts showed a less distinct gradient with depth; however, deeper layers still exhibited a greater contribution of protein-like components (e.g. C9), while surface layers were characterized by higher degrees of humification (Figs. 5b and 6). Finally, the WSOM extracted using Aqua dest. exhibited significantly greater variability across samples compared to K_2_SO_4_ extracts (Fig. 7).

## Discussion

We compared two WSOM extraction methods—use of 0.5 M K_2_SO_4_ and deionized water—to evaluate their impact on dissolved organic carbon (DOC) concentration and DOM composition across different soils and depth profiles. Soil samples (217 samples taken from 83 soil cores) were obtained from four central European regions: the Tyrolean Alps (Austria), and the Ore Mountains, Sauerland, and Black Forest (Germany). While WSOM represents only a small portion of soil OM, the presence, relative composition, and quantity of different WSOM fractions are indicative of soil biogeochemical processes. For instance, protein-like fluorescence has previously been linked to DOM bioavailability in aquatic systems^38^, and high BIX has been found to be indicative of recent autochthonous contribution of DOM^32^. Using absorbance and fluorescence spectroscopy, including PARAFAC-resolved components, we elucidate the impact of extractant choice on indicators of soil carbon biochemical reactivity/processing potential (WSOM composition)—as inferred by different representative DOM optical indices and fluorescent signatures in soil water extracts. Our findings directly inform soil extractant selection and the interpretation of DOC concentration and DOM composition analysis results, with implications for our understanding of catchment DOM dynamics and processes.

### Compositional variability between extraction methods

Our results highlight contrasting fluorescence and optical properties of soil extracts obtained via the two extraction methods. Aqua dest. extracts showed greater compositional variability across samples, and consistently exhibited elevated SUVA_254_, FI, BIX, and a300 values—indicating a higher proportion of aromatic, microbially influenced, and chromophoric WSOM. Additionally, Aqua dest. extractions revealed stronger contributions from protein-like PARAFAC components - often associated with fresh, labile organic matter and microbial by-products. In contrast, K_2_SO_4_ extractions—despite yielding higher DOC concentrations—were more compositionally consistent and characterized by humic-like fluorescence, suggesting the preferential desorption of older, mineral-bound WSOM fractions in salt extracts.

These differences likely reflect the contrasting chemistries of the extraction solutions. Aqua dest., with its low ionic strength, better solubilizes fresh and biologically derived compounds, consistent with observations from^20,43^—that water extractions often target hydrophilic, low-molecular-weight DOM fractions, including proteinaceous substances that may be lost or precipitated in high-salt conditions^44^. The higher variability (e.g., increased coefficient of variation) observed in Aqua dest. extracts are likely a result of the dynamic nature of these labile compounds, which are sensitive to spatial and temporal environmental fluctuations in microbial activity and recent organic inputs^4^.

Elevated salt concentrations in K_2_SO_4_ extracts may influence DOM optical properties. Increased ionic strength has been shown to promote molecular coiling of humic-like DOM, suppress ionization, and induce fluorescence quenching effects comparable to low-pH conditions^45^, potentially reducing the fluorescence intensity of certain humic-like fluorophores. However, in this study, humic-like PARAFAC components showed higher contributions in the K_2_SO_4_ extracts, indicating that salt-induced quenching of humic fluorophores is unlikely to be the dominant driver of the observed differences. Nevertheless, such quenching effects may still occur to some extent and cannot be entirely excluded, even if they are not directly resolved by our measurements.

Water extraction may inflate SUVA_254_ values due to the lack of flocculation of fine particles and colloids, which can enhance light scattering and artificially increase absorbance at 254 nm^12^. Consistent with this mechanism, we observed comparatively high SUVA_254_ values in our Aqua dest. extracts. While these values may result from elevated absorbance at 254 nm combined with lower DOC concentrations, scattering effects cannot be excluded and likely contribute to the observed SUVA_254_ patterns, as previously reported^12^.

In contrast, the higher ionic strength of K_2_SO_4_ promotes cation exchange and competitive desorption processes, which can disrupt metal-organic complexes and release more stabilized humic substances bound to mineral surfaces^5,12^. This results in a relatively larger proportion of DOC recovery, but markedly lower compositional variability of sample extracts (as observed in our study) using this method.

Finally, salt-induced protein precipitation (“salting-out”) provides an additional explanation for the observed patterns. Salts such as K_2_SO_4_ can reduce protein solubility, thereby suppressing the recovery of protein-like DOM components in soil extracts^44^. This mechanism likely contributes to the stronger protein-like signals observed in Aqua dest. extracts and the very low intensities of typical protein-like PARAFAC components (e.g., C4 and C7) in K_2_SO_4_ extracts (Fig. 5).

### Comparison with previous studies

Our findings not only align with previous studies, but importantly advance beyond them. Consistent with other studies^6,20,43,44^, we found that water-based extractions predominantly capture relatively more labile, hydrophilic DOM (as indicated by higher contributions of protein-like fluorescence and higher BIX, FI, and S_R_), whereas salt solutions can mobilize mineral-associated, humified pools - as reflected in a higher contribution of humic-like fluorescence.

An earlier study^12^ reported a strongly fluorescing band at 240 nm excitation in Aqua dest. extracts—but notably absent in several salt solutions, including K_2_SO_4_—being attributed to either an artefact or amino acid fluorescence from leached WSOM. The aforementioned study further demonstrated that salt extraction selectively suppresses fluorescence of reduced humic DOM, whereas more oxidized humic fractions remain largely unaffected. In contrast, protein-like fluorescence was consistently enhanced in Aqua dest. extracts. This pattern is consistent with evidence that increased salinity can induce fluorescence quenching^45^, preferentially affecting specific, salt-sensitive DOM fractions and thereby altering observed spectral characteristics in salt extracts.

A key advancement of our study is the demonstration that Aqua dest. extracts exhibit more pronounced protein-like PARAFAC components and greater sample-to-sample variability. This suggests that water extractions are particularly sensitive to short-term or localized changes in fresh organic inputs and microbial activity. In contrast, salt extractants yielded higher overall DOC recovery but displayed lower variability in fluorescent signatures, limiting their ability to capture DOM compositional diversity among samples. These points echo methodological concerns raised previously^20^ about biases in soluble protein recovery, but they also underscore an underappreciated implication: the choice of extractant substantially influences DOM characterization and, consequently, the ecological interpretation of soil carbon processes.

### Depth patterns in DOC concentration

DOC concentrations typically decreased with depth, regardless of extraction method. This pattern reflects the general decline in organic carbon availability deeper in the soil profile. This interpretation is supported by many studies of lowland and temperate soils, which show a clear decline of DOC with depth linked to reduced inputs and enhanced degradation processes^4,46,47^. Our Austrian Alpine sampling sites exhibited comparatively uniform and low DOC concentrations throughout their profile. This observation may reflect the nature of characteristic Leptosols for this region–having only a thin sediment layer above (fractured) bedrock material, facilitating rapid subsurface flow through a dense network of fractures, with rapid removal of DOC with depth through interaction with mineral surfaces and microbial consumption (attenuation processes). Such site-specific hydrological controls highlight that vertical DOC patterns are not universal, but depend on local soil structure and flow regimes. However, the observed lack of trend may also be influenced by the low sample size in the upper soil layers of this region. To further elucidate how the observed depth patterns map onto composition, we compared WSOM composition with depth for both extraction methods.

### Depth patterns in WSOM composition

Our results reveal consistent depth-related changes in WSOM composition, with Aqua dest. extractions showing a pronounced, but also more variable, shift from humic-like substances in surface layers to protein-like, microbially derived components at depth, when compared to K_2_SO_4_ extractions. In the Ore Mountains and Sauerland, we observed an initial spike in protein-like fluorescence in the surface layer, likely reflecting fresh DOM inputs from vegetation. Consistent with this, biological contributions decreased with depth. This pattern aligns with earlier findings by^12,16,21^, who observed similar compositional gradients in WSOM across soil profiles. Further, studies utilizing biomarkers and high–resolution mass spectrometry have demonstrated that microbial–derived compounds and necromass become more prominent in deeper soil horizons, where fresh organic inputs are limited^48,49^ and old microbially processed compounds dominate^5^. In contrast, in the Tyrolean Alps we could not detect a clear depth-related pattern in WSOM composition (similar to DOC concentration).

The clearer and more variable gradient captured by Aqua dest. likely reflects its ability to solubilize more labile, hydrophilic DOM fractions^43^ which may have recently been produced or transformed by microorganisms. In contrast, K_2_SO_4_ preferentially extracts more stabilized, mineral-bound compounds. WSOM systematically changes with depth, and extraction protocols influence how clearly these gradients are resolved, impacting their interpretation. We found water-based extraction better suited to track microbial activity and recent organic matter inputs (e.g. using indices such as BIX, FI, S_R_ and protein-like DOM) along depth profiles, whereas salt-based extractions are better suited for capturing larger shares of certain stable WSOM pools (e.g. maximizing the DOC extraction and humic-like fractions). These findings have important implications for comparing WSOM across sites and time. To better evaluate ecological relevance, we recommend that future studies directly compare extraction results with soil pore water, which would help identify which method most accurately reflects natural DOM mobilization and fluxes, ultimately improving assessments of carbon cycling and the role of WSOM in linking terrestrial and aquatic ecosystems.

### Implications for soil carbon cycling

Our results clearly demonstrate that the choice of soil extraction method profoundly shapes both the concentration and composition of WSOM in soil extracts. K_2_SO_4_ extractions consistently yielded higher DOC concentrations dominated by humic-like fluorescence, reflecting a more stabilized and persistent WSOM pool. In contrast, deionized water (Aqua dest.) extracts exhibited greater compositional variability, enriched in protein-like, microbially derived DOM, and captured depth–dependent shifts as well as the more dynamic, labile fractions of WSOM.

These differences, consistent across four different catchment soils in Germany and Austria, are ecologically meaningful. This is because soil organic matter is not static—and can vary seasonally^22,50^, and can undergo rapid transformations during decomposition, or persist for millennia^51^, depending on its composition and complex interactions between hydrological, physicochemical and ecological factors^4,5,52^ (e.g. temperature, oxygen and nutrient concentration and mobility in soil).

Water extracts reveal compositional complexity tied to temporal and spatial environmental fluctuations, highlighting short-term dynamics, microbial activity, and recent organic matter inputs. In contrast, K_2_SO_4_ extracts provide a broader, more consistent signal, reflecting mineral-associated, humified, and long-term WSOM pools.

This distinction has practical implications for research: selecting an appropriate extraction method depends on the study goal. We found that Aqua dest. recovered DOM was characteristically more soluble, responsive to recent inputs, and likely to be mobilized during runoff or subsurface flow, fuelling microbial respiration in downstream aquatic systems. Contrastingly, K_2_SO_4_-recovered fractions were found to be less soluble and more stable, and characteristic of long-term terrestrial carbon reservoirs rather than immediate inputs to aquatic ecosystems. Recognizing which WSOM fractions are captured is therefore critical when comparing datasets across sites, assessing microbial processing, or linking soil carbon turnover to downstream carbon fluxes.

Overall, our study highlights that extraction method choice is not merely technical, but fundamentally shapes ecological interpretation of soil carbon pools and DOM dynamics. Future studies should explicitly consider these methodological sensitivities when designing experiments, interpreting results, or integrating WSOM into catchment-scale carbon models under changing climate or land-use conditions.

## Data Availability

The datasets used and/or analysed during the current study available from the corresponding author on reasonable request.

## References

[1] Batjes, N. H. Total carbon and nitrogen in the soils of the world. *Eur. J. Soil. Sci.* **47** (2), 151–163 (1996).

[2] Schlesinger, W. H. & Andrews, J. A. Soil respiration and the global carbon cycle. *Biogeochemistry* **48**, 7–20 (2000).

[3] Lehmann, J. & Kleber, M. Perspective the contentious nature of soil organic matter. *Nature*. 1–9. 10.1038/nature16069 (2015). 10.1038/nature16069 26595271

[4] Kalbitz, K., Solinger, S., Park, J. H., Michalzik, B. & Matzner, E. Controls on the dynamics of dissolved organic matter in soils: A review. *Soil. Sci.* **165** (4), 277–304 (2000).

[5] Kaiser, K. & Kalbitz, K. Cycling downwards–dissolved organic matter in soils. *Soil. Biol. Biochem.* **52**, 29–32 (2012).

[6] Herbert, B. E. & Bertsch, P. M. Characterization of dissolved and colloidal organic matter in soil solution: A review. *Carbon Forms Funct. Soils* 63–88 (1995).

[7] Ohno, T., Amirbahman, A. & Bro, R. Parallel factor analysis of excitation–emission matrix fluorescence spectra of water soluble soil organic matter as basis for the determination of conditional metal binding parameters. *Environ. Sci. Technol.* **42** (1), 186–192 (2008). 18350895 10.1021/es071855f

[8] Raymond, P. A. & Saiers, J. E. Event controlled DOC export from forested watersheds. *Biogeochemistry* **100** (1), 197–209. 10.1007/s10533-010-9416-7 (2010).

[9] Parlanti, E., Woerz, K., Geoffroy, M. & Lamotte, M. Dissolved organic matter fluorescence spectroscopy as a tool to estimate biological activity in a coastal zone submitted to anthropogenic inputs dissolved organic matter uorescence spectroscopy as a tool to estimate biological activity in a coastal zone. *Org. Geochem.* **31** (August 2016), 1765–1781. 10.1016/S0146-6380(00)00124-8 (2000).

[10] McKnight, D. M. et al. Spectrofluorometric characterization of dissolved organic matter for indication of precursor organic material and aromaticity. *Limnol. Oceanogr.* **46** (1), 38–48 (2001).

[11] Boodoo, K. S., Fasching, C. & Battin, T. J. Sources transformation, and fate of dissolved organic matter in the gravel bar of a prealpine stream. *J. Geophys. Res. Biogeosci.* **125** (8), (2020). e2019JG005604.

[12] Gabor, R. S. et al. Influence of leaching solution and catchment location on the fluorescence of water-soluble organic matter. 10.1021/es504881t (2015). 10.1021/es504881t 25671820

[13] Hassouna, M., Massiani, C., Dudal, Y., Pech, N. & Theraulaz, F. Changes in water extractable organic matter (WEOM) in a calcareous soil under field conditions with time and soil depth. *Geoderma*. **155** (1–2), 75–85 (2010).

[14] Sun, H. Y. et al. Water-extractable organic matter and its fluorescence fractions in response to minimum tillage and organic farming in a cambisol. *Chem. Biol. Technol. Agric.* **4** (1), 15 (2017).

[15] Deng, Y., Boodoo, K. S., Knorr, K. & Glatzel, S. Assessing the impact of land use on peat degradation in bogs in the Enns Valley, Austria. *Soil. Use Manag* **41** (1), e70013. (2025).

[16] Kalbitz, K. Properties of organic matter in soil solution in a German fen area as dependent on land use and depth. *Geoderma* **104** (3–4), 203–214 (2001).

[17] Jones, D. L. & Willett, V. B. Experimental evaluation of methods to quantify dissolved organic nitrogen (DON) and dissolved organic carbon (DOC) in soil. *Soil. Biol. Biochem.* **38** (5), 991–999 (2006).

[18] Ghani, A., Dexter, M. & Perrott, K. W. Hot-water extractable carbon in soils: A sensitive measurement for determining impacts of fertilisation, grazing and cultivation. *Soil. Biol. Biochem.* **35** (9), 1231–1243 (2003).

[19] Füleky, G. & Czinkota, I. Hot water percolation (HWP): A new rapid soil extraction method. *Plant. Soil.* **157** (1), 131–135 (1993).

[20] Greenfield, L. M., Hill, P. W., Paterson, E., Baggs, E. M. & Jones, D. L. Methodological bias associated with soluble protein recovery from soil. *Sci. Rep.* **8** (1), 11186 (2018). 30046143 10.1038/s41598-018-29559-4 PMC6060134

[21] Corvasce, M., Zsolnay, A., D’Orazio, V., Lopez, R. & Miano, T. M. Characterization of water extractable organic matter in a deep soil profile. *Chemosphere* **62** (10), 1583–1590 (2006). 16171846 10.1016/j.chemosphere.2005.07.065

[22] Schiedung, H. & Bornemann, L. Seasonal variability of soil organic carbon fractions under arable land. *Pedosphere* **27** (2), 380–386 (2017).

[23] Zhou, P. et al. Spatial patterns and environmental functions of dissolved organic matter in grassland soils of China. *Nat. Commun.* **15** (1), 6356 (2024). 39069514 10.1038/s41467-024-50745-8 PMC11284229

[24] Esri World Imagery [Basemap]. https://www.esri.com/en-us/arcgis/products/arcgis-online/basemap-imagery (2025).

[25] Sun, T., Dong, L., Mao, Z. & Li, Y. Fine root dynamics of trees and understorey vegetation in a chronosequence of betula platyphylla stands. *Ecol. Manage.* **346**, 1–9 (2015).

[26] Singer, G. et al. Biogeochemically diverse organic matter in alpine glaciers and its downstream fate. *Nat. Geosci.* **5** (10), 710–714. 10.1038/ngeo1581 (2012).

[27] Weishaar, J. et al. Evaluation of specific ultra-violet absorbance as an indicator of the chemical content of dissolved organic carbon. *Environ. Sci. Technol.* **37** (20), 4702–4708. 10.1021/es030360x (2003). 14594381 10.1021/es030360x

[28] Spencer, R. G. M. et al. Chromophoric dissolved organic matter export from U.S. rivers. *Geophys. Res. Lett.* **40** (8), 1575–1579. 10.1002/grl.50357 (2013).

[29] Helms, J. R. et al. Absorption spectral slopes and slope ratios as indicators of molecular weight, source, and photobleaching of chromophoric dissolved organic matter. *Limonology Oceanogr.* **53** (3), 955–969. 10.4319/lo.2008.53.3.0955 (2008).

[30] Pucher, M. et al. StaRdom: Versatile software for analyzing spectroscopic data of dissolved organic matter in R. *Water* **11** (11), 2366 (2019).

[31] Ohno, T. Fluorescence inner-filtering correction for determining the humification index of dissolved organic matter. *Environ. Sci. Technol.* **36** (4), 742–746 (2002). 11878392 10.1021/es0155276

[32] Huguet, A. et al. Properties of fluorescent dissolved organic matter in the gironde estuary. *Org. Geochem.* **40** (6), 706–719. 10.1016/j.orggeochem.2009.03.002 (2009).

[33] R Core Team. R: A Language and Environment for Statistical Computing. *R Found. Stat. Comput. Vienna Austria*. https://doi.org/http://www.R-project.org/ (2024)

[34] Murphy, K. R., Stedmon, C. A., Wenig, P. & Bro, R. OpenFluor—an online spectral library of auto-fluorescence by organic compounds in the environment. *Anal. Methods*. **6** (3), 658–661 (2014).

[35] Coble, P. G. Characterization of marine and terrestrial DOM in seawater using excitation-emission matrix spectroscopy. *Mar. Chem.* **51** (4), 325–346. 10.1016/0304-4203(95)00062-3 (1996).

[36] Kim, J., Song, B. C. & Kim, T. H. Origin of dissolved organic carbon under phosphorus-limited coastal-bay conditions revealed by fluorescent dissolved organic matter. *Front. Mar. Sci.* **9**, 971550 (2022).

[37] Lambert, T., Bouillon, S., Darchambeau, F., Massicotte, P. & Borges, A. V. Shift in the chemical composition of dissolved organic matter in the Congo River Network. *Biogeosciences* **13** (18), 5405–5420 (2016).

[38] Fellman, J. B., Hood, E., Edwards, R. T. & D’Amore, D. V. Changes in the concentration, biodegradability, and fluorescent properties of dissolved organic matter during stormflows in coastal temperate watersheds. *J. Geophys. Res. Biogeosci*. **114** (1), 1–14. 10.1029/2008JG000790 (2009).

[39] Lambert, T. et al. Effects of human land use on the terrestrial and aquatic sources of fluvial organic matter in a temperate river basin (The Meuse River, Belgium). *Biogeochemistry* **136** (2), 191–211 (2017).

[40] Maurischat, P. et al. The Glacial–Terrestrial–Fluvial Pathway: A Multiparametrical Analysis of Spatiotemporal Dissolved Organic Matter Variation in Three Catchments of Lake Nam Co, Tibetan Plateau. *Sci. Total Environ.* **838**, 156542 (2022). 35690211 10.1016/j.scitotenv.2022.156542

[41] Murphy, K. R. et al. Organic matter fluorescence in municipal water recycling schemes: Toward a unified PARAFAC model. *Environ. Sci. Technol.* **45** (7), 2909–2916 (2011). 21361278 10.1021/es103015e

[42] Bouchachi, N., Obernosterer, I., Marie, B., Crispi, O. & Ortega-Retuerta, E. Phosphorus limitation determines the quality of dissolved organic matter released by marine heterotrophic prokaryotes. *Limnol. Oceanogr. Lett.* **8** (2), 330–339 (2023).

[43] Zsolnay, Á. Dissolved organic matter: Artefacts, definitions, and functions. *Geoderma* **113** (3–4), 187–209 (2003).

[44] Shih, Y., Prausnitz, J. M. & Blanch, H. W. Some characteristics of protein precipitation by salts. *Biotechnol. Bioeng.* **40** (10), 1155–1164 (1992). 18601066 10.1002/bit.260401004

[45] Provenzano, M. R., Cilenti, A., Gigliotti, G. & Senesi, N. Spectroscopic investigation on hydrophobic and hydrophilic fractions of dissolved organic matter extracted from soils at different salinities. *CLEAN–Soil Air Water*. **36** (9), 748–753 (2008).

[46] Kögel-Knabner, I. The Macromolecular organic composition of plant and microbial residues as inputs to soil organic matter. *Soil. Biol. Biochem.* **34** (2), 139–162 (2002).

[47] Michalzik, B., Kalbitz, K., Park, J. H., Solinger, S. & Matzner, E. Fluxes and concentrations of dissolved organic carbon and nitrogen–a synthesis for temperate forests. *Biogeochemistry*. **52** (2), 173–205 (2001).

[48] Lorenz, M., Hofmann, D., Steffen, B., Fischer, K. & Thiele-Bruhn, S. The molecular composition of extractable soil microbial compounds and their contribution to soil organic matter vary with soil depth and tree species. *Sci. Total Environ.* **781**, 146732 (2021).

[49] Liang, C., Amelung, W., Lehmann, J. & Kästner, M. Quantitative assessment of microbial necromass contribution to soil organic matter. *Glob Chang. Biol.* **25** (11), 3578–3590 (2019). 31365780 10.1111/gcb.14781

[50] Embacher, A., Zsolnay, A., Gattinger, A. & Munch, J. C. The dynamics of water extractable organic matter (WEOM) in common arable topsoils: I. Quantity, quality and function over a three year period. *Geoderma* **139** (1–2), 11–22 (2007).

[51] Schmidt, M. W. I. et al. Persistence of soil organic matter as an ecosystem property. *Nature*. **478** (7367), 49–56. 10.1038/nature10386 (2011). 21979045 10.1038/nature10386

[52] Guggenberger, G. & Kaiser, K. Dissolved organic matter in soil: Challenging the paradigm of sorptive preservation. *Geoderma*. **113** (3–4), 293–310 (2003).

